# Mammogram adherence after a rise in telehealth use: a longitudinal study of a large health system

**DOI:** 10.1016/j.pmedr.2025.103320

**Published:** 2025-11-23

**Authors:** Ofer Amram, Pablo Monsivais, Jeanne Robison, Chaya Mangel Pflugeisen, Namrata Sanjeevi

**Affiliations:** a Elson S. Floyd College of Medicine, Washington State University, Spokane, WA 99202, USA; b Department of Nutrition and Exercise Physiology, Elson S. Floyd College of Medicine, Washington State University, Spokane, WA 99202, USA; c Paul G. Allen School for Global Animal Health, Washington State University, Pullman, WA 99164, USA; d MultiCare Institute for Research & Innovation, Tacoma, WA 98415, USA; e MultiCare Deaconess Cancer & Blood Specialty Centers Spokane Washington, 99204, USA

**Keywords:** Mammography, Telehealth, Rurality, Breast Cancer, Prevention

## Abstract

**Objective:**

Early detection of breast cancer through routine screening improves survival. Increased engagement with healthcare services may promote mammography uptake. This study examined whether greater telehealth use is associated with higher mammogram screening rates.

**Methods:**

We analyzed data from January 1st 2018 to December 31st 2023 from the MultiCare Health System in Washington State. For each year, we determined whether a patient received a screening mammogram during that year or the following year. We assessed the association between type of encounter (≥1 telehealth visit vs. in-person only vs. no encounters) and the likelihood of having a mammogram, adjusting for race/ethnicity, insurance type, age, and pre−/post-COVID-19 period.

**Results:**

Among 140,390 female patients (609,061 patient-year observations), those with no encounters were least likely to undergo mammography. Women who used telehealth were less likely to be screened than those with in-person visits but more likely than those with no visits (rural: Odds Ratio (OR) = 0.50, 95 % Confidence Interval (CI) = 0.43,0.60; urban: OR = 0.68, 95 % CI = 0.65,0.70). Telehealth rose from 0.04 % to 4 % post-COVID-19, while mammography rates increased from 0 % to an average of 13 %.

**Conclusions:**

Expanding telehealth access may increase routine mammography, particularly among previously unscreened populations.

## Highlights

• Women with at least one telehealth visit had higher odds of receiving screening mammograms.
• The link between telehealth use and mammogram uptake was stronger in urban areas.
• Telehealth expansion to deliver primary care may result in earlier cancer detection

## 1 Introduction

Breast cancer is the most common cancer diagnosed in women, with an average annual rate of 125 cases per 100,000 (*American Cancer Society. Cancer Facts and Figures,* 2020). However, over the last 30 years, breast cancer death rates have declined from 32 per 100,000 women in 1992 to 18.8 per 100,000 women in 2022 (*National Cancer Institute. Cancer Stat Facts: Female Breast Cancer*, 2021), with a 5-year relative survival of 91.2 % in 2014–2020 (*National Cancer Institute. Cancer Stat Facts: Female Breast Cancer*, 2021). Increasing survival rates have been attributed to more effective treatment options, but also to earlier detection among women who participate in routine breast cancer screening. While a small percentage of women with above average risk may be considered for screening before age 50, the general recommendation is to offer regular screening mammograms to all women by age 50 (*National Center for Health Statistics. Mammography*, 2025; Siu and on behalf of the U.S. Preventive Services Task Force, 2016).

Screening mammograms play a key role in the early detection of breast cancer (*Centers for Medicare, and Medicaid Services. Medicare National Coverage Determinations Manual Chapter 1, Part 4 (Sections 200–310.1) Coverage Determinations,* 2025). The consensus recommendation is that bi-annual screening is the minimum frequency for health maintenance among women with average risk (Budh and Sapra, n.d.). Despite these guidelines, disparities in the use of mammograms exist, such that screening rates are disproportionately lower among women who are non-White, uninsured, and from rural communities (*American Cancer Society. Breast Cancer Facts and Figures 2019–2020,* 2025; Henley and Jemal, 2018). Consistently, despite the overall decline, breast cancer mortality remains persistently higher in racial/ethnic minorities (Blackman and Masi, 2006).

Telehealth may facilitate access to screening services by increasing engagement with health care in general and by referral to screening services (McGrowder et al., 2021),. (*Health Resources and Services Administration. What is Telehealth?,* 2025) The Centers for Disease Control and Prevention has identified telehealth as a promising public health tool because it has the potential to improve access to care, especially for underserved populations and residents of rural areas (*Centers for Disease Control and Prevention. Research Anthology: Telehealth and Telemedicine. Available from*, 2025). Although telehealth has the potential to dramatically improve access to breast cancer screening, research has shown inequities in utilization of telehealth services, particularly for those who are already underserved (Zhang et al., 2024). With the rapid growth of telehealth services during the COVID-19 pandemic and the likelihood of these services remaining in use and expanding overtime, there is a need to understand how these services could influence use of screening mammograms.

This study utilizes data from a large clinical network in Washington State to explore telehealth and in-person interactions with the healthcare system and their association with mammogram uptake among women aged 50 and older. More specifically, the study aims to examine whether telehealth use is linked to increased screening mammography, with a particular focus on its impact within underserved populations. We hypothesize that telehealth engagement will be associated with higher rates of mammogram screenings, especially for women residing in rural areas where healthcare access can be more limited.

## 2 Methods

### 2.1 Study design and population

The population for this study included patients at the MultiCare Health System. MultiCare is the largest not-for-profit, community-based health care organization in Washington State with more than 20,000 team members, including employees, providers and volunteers (*About MultiCare. MultiCare*, 2025). MultiCare represents a comprehensive system of healthcare services in locations across the State of Washington and includes inpatient, primary, virtual, urgent, and dedicated pediatric and specialty care services. MultiCare offers cancer care services at multiple locations across Washington State, both in rural and urban areas. In 2024, Washington State's population was over 7.5 million with 11.5 % of the population living at or below the poverty line (Washington Population, 2024). In 2024, 69.9 % of the population was non-Hispanic White, 9.2 % Asian and 3.9 % African American. In terms of health coverage, 49.4 % of the population participates in employee provided plans, 17.7 % receives benefits through Medicaid, 10.9 % through Medicare, 13.4 % through non-group plans, and 2.5 % receive military or Veterans Affairs health benefits (*Washington. Data USA*, 2025). The study's protocol was approved by the Institutional Review Board of MultiCare, the data holder.

### 2.2 Data structure and selection

The MultiCare Health System data analyzed for this study comprised in-person or telehealth encounters by women, aged 50 years or older within primary care or women's health/gynecology practices. Only visits from January 2018 through December 2023 were included. Correspondingly, only women with at least one of these qualifying encounters were included in the study. Screening mammograms were identified based on procedure codes and their accompanying descriptions; codes explicitly labeled as “screening” were classified accordingly, and those without clear labels were reviewed manually and categorized as screening or diagnostic based on the description provided.

### 2.3 Measures

#### 2.3.1 Outcome

To allow for a 1-year interval between a woman participating in a health care encounter and then completing a screening mammogram, year-level data accounted for screening mammograms in the specific visit year and the following calendar year.The number of screening mammograms in a specific encounter year (i.e., the year during which an encounter was recorded) and the following calendar year was computed, based on which year-level mammogram data were coded as having 1) no screening mammograms or 2) at least 1 screening mammogram (Yes, vs. No). For example, the number of screening mammograms in both 2018 and 2019 were used to compute mammogram data for the 2018 encounter year. Similarly, the number of screening mammograms in 2022 and 2023 were used to represent mammogram data for the year, 2022. Since the study period was up to December 2023, mammogram data were calculated in this manner for encounter years 2018 through 2022. In the dataset, 13 women had more than 1 screening mammogram in a given year.

#### 2.3.2 Explanatory variable

For all women included in the study, encounters were summarized at the year-level and only records with <4 annual encounters were included. Women with ≥4 annual encounters were excluded as higher health care use could potentially indicate presence of chronic health conditions. Year-level encounters were then coded into one of the following three categories for each patient encounter year within the health care system: 1) no encounters; 2) only in-person encounters; or 3) at least 1 telehealth encounter (this group includes women with only telehealth encounters as well as those with telehealth and in-person encounters). To test our hypothesis that women in rural areas would benefit more from telehealth interaction, we measured urbanicity of residence using Rural-Urban Commuting Area Codes classification (RUCA), with Zip codes coded with RUCA 1 and 2 considered urban while the rest (3 or greater) were considered Suburban/Rural (Urban, yes vs. no). To assess whether rurality modifies the relationship of encounter type with having a mammogram, an interaction variable with six levels was included: ***in-person-urban***: years where women had an in-person visit only and resided in urban areas (Reference Level); ***in-person-rural:*** years where women had an in-person visit only and resided in rural areas; ***telehealth-urban***: years where women had at least one telehealth visit and resided in urban areas; ***telehealth-rural***: years where women had at least one telehealth visit and resided in rural areas; ***no visit-urban***: years where women had no interaction with MultiCare and resided in urban areas; and ***no visit-rural***: years where women had no interaction with MultiCare and resided in rural areas.

Sociodemographic data included patients' race and ethnicity (Hispanic, Non-Hispanic White, Non-Hispanic Black, Non-Hispanic Asian, American Indian Alaskan Native (AI/AN) and others) and age at the time of most recent encounter (50–60, 60–70 and over 70 years old). Insurance coverage for each encounter year was coded as having 1) only encounters paid by commercial insurance; 2) at least one Medicaid-paid encounter (but not Medicare, other government insurance or self-paid encounters); 3) at least one Medicare-paid encounter (but not other government insurance or self-paid encounters); 4) at least one other government insurance-paid encounter (but not self-paid encounters); 5) at least one self-paid encounter; and 6) encounters paid by other non-government insurance. Finally, we also used Area Deprivation Index (ADI) for each patient's most recent Zip Code, and timing of encounter (pre-COVID (before 2020) or post-COVID (2020 and beyond)) as covariates.

### 2.4 Statistical analysis

Chi-square tests were conducted to examine differences in patient-level demographic characteristics by the type of encounter. A logistic multi-level generalized linear model (Patient level), including the derived interaction variable capturing the relationship between encounter type and urbanicity, examined the association of encounter type and urbanicity with uptake of screening mammograms. Stratified analyses utilizing logistic multi-level generalized linear model were conducted to examine the association of encounter type (in-person vs. telehealth vs no visit) with mammogram uptake for each level of urbanicity.

We additionally subsetted the data to include only women with at least one visit during pre-COVID (i.e., 2018 and 2019), but had no mammography during this period (*n* = 19,522). Descriptive statistics were used to examine encounter patterns and mammography uptake rates in this group of women during post-COVID (i.e., 2020 and beyond). Analyses were conducted using R (version 4.3.1).

## 3 Results

There were 140,390 female patients in the study; of these, 392 had breast cancer, accounting for a total of 609,061 patient-year observations from 2018 to 2022(Table 1). About 63 % of the women had at least one mammogram during the study period. About 95 % of the women resided in urban areas, with about 38 % over the age of 70, the largest age group. In terms of health coverage, 54.4 % had at least one encounter covered by Medicare, and 33.4 % had commercial insurance. The majority of patients were non-Hispanic white, followed by non-Hispanic Asian (84.0 % and 4.9 %, respectively). Telehealth was utilized at least once by 25.5 % of the patients, while the remaining 74.5 % of patients exclusively attended in-person visits. Telehealth users were significantly more likely to be living in suburban/rural areas, younger, and non-Hispanic White, while non-telehealth users were more likely to be older, covered by Medicare, and Asian.

**Table 1:** Demographic characteristics of female patients (*N* = 140,390) aged 50 years or older with encounters in primary care or women's health/gynecology practices, by encounter type, based on data from 2018 to 2023 collected from the MultiCare Health System in Washington State.^a^, ^b^, ^c^

	Overall (*n* = 140,390)	In-Person encounters only (*n* = 104,527)	≥1 Telehealth encounter (*n* = 35,863)	p-value^d^
**Urbanicity**				
Urban (i.e., RUCA1 in (1,2))	134,003 (95.5)	99,953 (95.6)	34,050 (94.9)	<0.01
Suburban/Rural (RUCA 3–8)	6387 (4.6)	4574 (4.4)	1813 (5.1)	
**Age at time of first encounter**				
≥70 years	52,792 (37.6)	41,670 (39.9)	11,122 (31.0)	
60–70 years	43,472 (31.0)	31,422 (30.1)	12,050 (33.6)	<0.01
50–60 years	44,126 (31.4)	31,435 (30.1)	12,691 (35.4)	
**Coverage type**				
Only commercial insurance	46,816 (33.4)	33,845 (32.5)	12,423 (36.2)	
At least 1 Medicaid-paid encounter^e^	10,294 (7.4)	7368 (7.1)	2932 (8.2)	
At least 1 Medicare-paid encounter^f^	76,210 (54.4)	58,281 (55.9)	19,402 (50.1)	
At least 1 encounter paid by other government insurance^g^	2886 (2.1)	1936 (1.9)	1010 (2.7)	
At least 1 self-paid encounter	3843 (2.7)	2799 (2.7)	1209 (2.9)	
Other non-government insurance	2 (0.0)	2 (0.0)	0 (0.0)	<0.01
**Race/ethnicity**				
Hispanic	3961 (2.8)	2984 (2.9)	977 (2.7)	
Non-Hispanic White	117,949 (84.0)	87,394 (83.6)	30,555 (85.2)	
Non-Hispanic Black	4982 (3.6)	3436 (3.3)	1546 (4.3)	<0.01
Hawaiian/Pacific Islanders	1098 (0.8)	815 (0.8)	283 (0.8)	
Non-Hispanic Asian	6855 (4.9)	5688 (5.4)	1167 (3.3)	
AI/AN	1061 (0.8)	742 (0.7)	319 (0.9)	
Other/unknown	4484 (3.2)	3468 (3.3)	1016 (2.8)	
**Mammography uptake for the study period**^h^				
No	52,184 (37.2)	40,687 (38.9)	11,497 (32.1)	<0.01
Yes	88,206 (62.8)	63,840 (61.1)	24,366 (67.9)	
**Number of encounters per year**	0.91 ± 0.79	0.85 ± 0.75	1.08 ± 0.86	<0.01

NH/PI, Native Hawaiian/Pacific Islander; AI/AN, American Indian/Alaska Native; RUCA, Rural-Urban Commuting Area Codes classification.

a Data are presented as n (%) or mean ± standard deviation.

b Since the percentages are rounded up, they may not always sum to 100 %.

c Only includes primary care/obgyn in-person encounters and telehealth encounters.

d Chi-square tests were used to test the difference in proportions.

e Does not include individuals with Medicare, other government insurance or self-paid encounters.

f Does not include individuals with other government insurance or self-paid encounters.

g Does not include individuals with self-paid encounters.

h Defined as having at least one mammogram.

Table 2 shows both unadjusted and adjusted models. The final adjusted model showed that, compared to years in which women in urban areas had an in-person-only interaction with MultiCare, women in rural areas who also had an in-person interaction were more likely to have a mammogram (Odds Ratio (OR) = 1.12, 95 % Confidence Interval (CI) = 1.01,1.24). In years in which women had no interaction with MultiCare they were by far the least likey to have a yearly mammogram, with those residing in rural area having lower odds (OR = 0.21, 95 % CI = 0.19,0.23) followed by women living in urban areas (OR = 0.26, 95 % CI = 0.26,0.27). In years in which women had at least one telehealth visit, the likelihood of having a mammogram the following year decreased compared to women who had an in-person visit, but increased compared to women who had no vists, with those residing in rural area having the lowest odds (OR = 0.50, 95 % CI = 0.43,0.60) followed by women living in urban area (OR = 0.68, 95 % CI = 0.65,0.70). During the pre-COVID years (prior to 2020), women were less likely to have a mammogram compared to post-COVID years (OR = 0.9, 95 % CI = 0.89,0.90). Finally, residing in areas with higher deprivation was associated with lower odds of having a mammogram (OR = 0.91, 95 % CI = 0.90,0.92).

**Table 2:** Unadjusted and adjusted odds ratios of having a yearly mammogram among 140,390 female patients (609,061 patient-year observations) aged 50 years or older with encounters in primary care or women's health/gynecology practices, between 2018 and 2023 at the MultiCare Health System in Washington State.

	**Unadjusted**		**Adjusted**	
*Predictors*	**Odds Ratios**	**CI**	**Odds Ratios**	**CI**
(Intercept)			**0**	**0.00, 0.00**
**Encounters with health services, by urbanicity**				
*In-person only-Urban*				
*In-person only-Rural*	**0.8**	**0.72, 0.88**	**1.13**	**1.01, 1.26**
*Telehealth-Urban*	**0.67**	**0.64, 0.70**	**0.65**	**0.62, 0.69**
*Telehealth-Rural*	**0.25**	**0.20, 0.30**	**0.51**	**0.41, 0.62**
*No visits-Urban*	**0.21**	**0.20, 0.21**	**0.22**	**0.22, 0.23**
*No visits-Rural*	**0.15**	**0.14, 0.17**	**0.23**	**0.21, 0.25**
**Pre-Covid**				
*No*				
*Yes*	**0.88**	**0.87, 0.89**	**0.9**	**0.89, 0.91**
**Race/Ethnicity**				
Non-Hispanic White				
Hispanic	**0.68**	**0.61, 0.75**	**0.96**	**0.87, 1.06**
AI/AN	**0.43**	**0.35, 0.52**	**0.58**	**0.47, 0.70**
Non-Hispanic Asian	**0.77**	**0.72, 0.83**	**0.81**	**0.75, 0.88**
Non-Hispanic Black	**0.75**	**0.68, 0.82**	**0.97**	**0.88, 1.06**
Hawaiian/Pacific Islanders	**0.38**	**0.31, 0.45**	**0.54**	**0.45, 0.66**
Other/bi-racial	**0.47**	**0.43, 0.51**	**0.64**	**0.58, 0.71**
**Age Group**				
50–59				
60–70	**2.39**	**2.29, 2.49**	**2.3**	**2.20, 2.41**
70+	**1.12**	**1.07, 1.16**	**1.06**	**1.01, 1.12**
**Insurance - Coverage Type**				
Commercial				
Medicare	**0.62**	**0.62, 0.62**	**0.6**	**0.58, 0.63**
Medicaid	**0.34**	**0.34, 0.34**	**0.35**	**0.33, 0.37**
Government	**1.03**	**1.03, 1.03**	**0.76**	**0.68, 0.85**
Self Pay	**0.39**	**0.39, 0.39**	**0.3**	**0.27, 0.33**
**Area Deprivation Index**^a^	**0.9**	**0.89, 0.91**	**0.91**	**0.90, 0.92**

NH/PI, Native Hawaiian/Pacific Islander; AI/AN, American Indian/Alaska Native; CI, Confidence Interval.

a Odds associated with 1 unit increase.

**Fig. 1:**
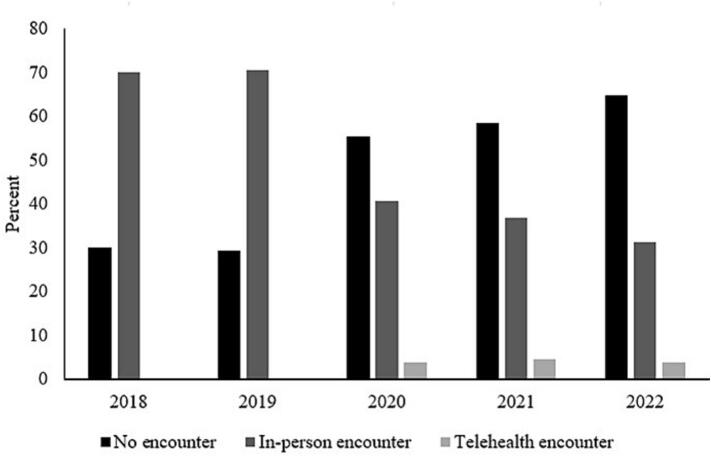
Encounter patterns among 19,522 (93,153 patient-year observations) female patients aged 50 years or older with at least one encounter and no mammography during the pre-COVID period, between 2018 and 2023 at the MultiCare Health System in Washington State.

**Fig. 2:**
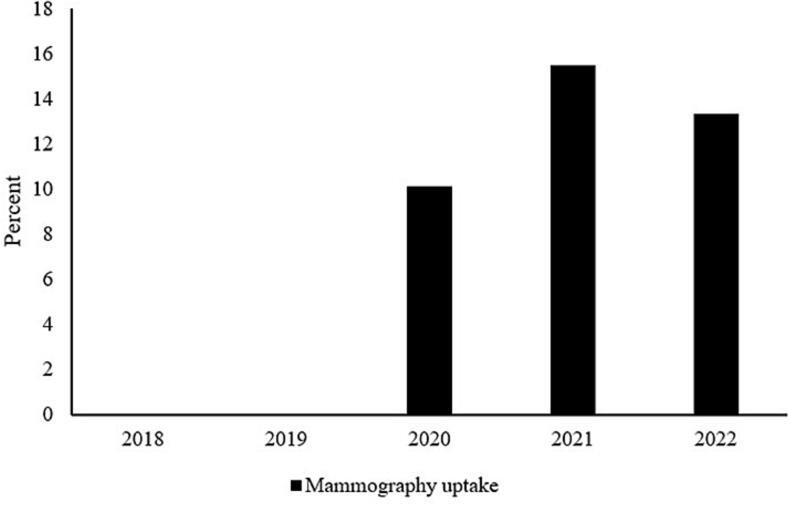
Mammography uptake rates among 19,522 (93,153 patient-year observations) female patients aged 50 years or older with at least one encounter and no mammography during the pre-COVID period, between 2018 and 2023 at the MultiCare Health System in Washington State.

Fig. 1, Fig. 2 indicate encounter pattern and mammography uptake rates among women with atleast one encounter and who had no mammography during pre-COVID. As shown in Fig. 1, about 4 % of women had at least 1 telehealth encounter during post-COVID years, whereas about 0.03 % and 0.05 % of women had atleast 1 telehealth encounter in 2018 and 2019, respectively. Further, in this group of women, mammography uptake rates increased from 0 % in 2018 and 2019 to about 10 %, 16 % and 13 % in 2020, 2021 and 2022, respectively (Fig. 2). The increase in mammography uptake among this group of women is also presented in Table 3.

**Table 3:** Adjusted odds ratios of having a yearly mammogram among 19,522 female patients (93,153 patient-year observations) aged 50 years or older with at least one encounter and no mammography during the pre-COVID period, between 2018 and 2023 at the MultiCare Health System in Washington State.

	**Adjusted**	
*Predictors*	*Odds Ratios*	*CI*
(Intercept)	0	0.00, 0.00
**Interaction with health services**		
In Person		
Atleast1Telehealth	1.54	1.20, 1.97
None	0.57	0.50, 0.66
**Urbanicity**		
Urban		
Rural	0.96	0.55, 1.68
**Area Deprivation Index**^a^	0.93	0.88, 0.99

NH/PI, Native Hawaiian/Pacific Islander; AI/AN, American Indian/Alaska Native; CI, Confidence Interval.

a Odds associated with 1 unit increase.

## 4 Discussion

In this study of women seeking healthcare within a large community hospital system in Washington State, having at least one telehealth encounter was associated with lower odds of screening mammogram uptake compared to exclusively in-person visits. However, compared to having no encounters at all, telehealth visits were significantly more advantageous, leading to higher odds of mammogram uptake. Notably, women with no healthcare encounters had the lowest likelihood of undergoing screening mammograms. Among women who used telehealth at some point during the post-COVID period, those with no healthcare encounters during pre-COVID showed an increase in mammography uptake. Further, the magnitude of this increase was greater than that found for women who had in-person encounters during pre-COVID. These findings highlight telehealth as a vital tool in facilitating access to essential health services. While in-person visits remain critical for improving mammogram screenings, telehealth offers a crucial alternative for reaching underserved populations, like women residing in rural areas. Telehealth may help reduce disparities in breast cancer screening and provide vital health maintenance and cancer screening coaching when in-person encounters are not possible.

The use of telehealth to conduct healthcare visits increased during the onset of the covid pandemic, peaked, and has declined in the last two years (*Peterson-KFF Health System Tracker. Outpatient telehealth use soared early in the COVID-19 pandemic but has since receded*, 2025; Demeke et al., 2021). Fortunately, the utilization of this additional tool for delivering health care has remained higher than in pre-covid years and, as this study shows, may improve uptake of cancer screening compared to having no encounters with providers. Some possible reasons for this relationship may be that telehealth may free up time and resources that would otherwise be spent on travel, child- or eldercare, or arranging time off work. This convenience can enable patients to save resources for essential in-person visits, such as screening mammograms. Telehealth may also allow opportunities for cancer screening education and mammogram reminders that would be unavailable to women who have barriers to in person visits.

An examination of demographic characteristics associated with telehealth utilization could provide an understanding of barriers related to use of this technology. In this study, we observed significant differences in demographic characteristics between women with only in-person encounters and those with at least 1 telehealth encounter. Notably, women aged 70 years or older had lower rates of having at least 1 telehealth encounter, a finding that is comparable with previous research, indicating that older adults were less likely to have access to telehealth capable technology (Pasquinelli et al., 2022). Consistent with another study (Bustamante et al., 2023), we also observed lower rates of telehealth utilization among women with at least one Medicare-paid encounter and non-Hispanic Asian women. In response to the pandemic, telehealth waivers were implemented in March 2020 that expanded Medicare coverage of telehealth services during the public health emergency (PHE) (KFF et al., n.d.). Subsequently, telehealth use increased substantially among Medicare beneficiaries during this period (Samson et al., 2025). While the use of pre-pandemic years in this study could explain the lower rates of telehealth use among women with Medicare-paid encounters, additional studies are warranted to examine telehealth coverage and use among Medicare beneficiaries during and beyond the PHE. Further, coverage by Medicare/Medicaid/other governmental insurance were significantly associated with lower odds of screening. The higher prevalence of reporting out-of-pocket payment for mammography among Medicare-only beneficiaries and uninsured women found in a previous investigation (Sabatino et al., 2019) could explain these patients' lower likelihood of screening and highlights the need to improve access to screening services. Significant disparities in uptake of screening mammograms by neighborhood deprivation were found, such that women from poorer neighborhoods had lower odds of screening. This finding is consistent with previous research (Kurani et al., 2020; Sprague et al., 2021) and reinstates the need to improve breast cancer screening in this group of women.

Finally, we found that women who were not engaged with the healthcare system routinely were less likely to have a mammogram. This result reinstates the importance of interactions with health care providers – in person or via telehealth – to improve breast cancer screening uptake. Exclusive use of in person visits was associated with a higher uptake of screening compared to telehealth use. More research is needed to understand if less health maintenance coaching is occurring during a telehealth visit than visits done in person or if those patients using telehealth also have greater barriers to cancer screening. Protocols for telehealth visits may consider incorporating cancer screening education and mammogram appointment reminders while the patient is in the virtual waiting room as a intervention to improve uptake among patients utilizing telehealth.

### 4.1 Study Limitations

This study has some limitations. First, the demographic profile of the sample is somewhat less diverse and represents a more affluent population than that of Washington State overall, as reflected by the area deprivation index distribution of the sample. Additionally, the data is limited to patient interactions within a single healthcare system, and we could not observe interactions with providers and clinics outside the system.

## 5 Conclusions

While there are barriers to delivering telehealth for both patients and providers, women may benefit from continued research and investment in addressing these barriers and continued support for improving technology at both the provider and patient interface levels. More research is needed to understand how telehealth providers conduct visits and its impact on health outcomes. Certainly, future healthcare policy supporting the expansion and use of telehealth to deliver primary care may result in earlier cancer detection and improved outcomes for cancer patients.

## Disclosure of funding and conflicts of interest

This study was funded by the Andy Hill CARE Fund. The authors have no conflicts of interest to declare.

## CRediT authorship contribution statement

**Ofer Amram:** Writing – original draft, Methodology, Formal analysis, Conceptualization. **Pablo Monsivais:** Writing – review & editing, Conceptualization. **Jeanne Robison:** Writing – review & editing. **Chaya Mangel Pflugeisen:** Writing – review & editing, Data curation. **Namrata Sanjeevi:** Writing – review & editing, Formal analysis.

## Declaration of competing interest

The authors declare the following financial interests/personal relationships which may be considered as potential competing interests: Ofer Amram reports financial support was provided by Andy Hill CARE Fund. If there are other authors, they declare that they have no known competing financial interests or personal relationships that could have appeared to influence the work reported in this paper.

## Data Availability

The data that has been used is confidential.
